# Sexual experience has no effect on male mating or reproductive success in house mice

**DOI:** 10.1038/s41598-019-48392-x

**Published:** 2019-08-21

**Authors:** Kerstin E. Thonhauser, Alexandra Raffetzeder, Dustin J. Penn

**Affiliations:** 1 Konrad Lorenz Institute of Ethology, Department of Interdisciplinary Life Sciences, University of Veterinary Medicine Vienna, Vienna, Austria; 2 Institute of Laboratory Animal Science, Department for Biomedical Sciences, University of Veterinary Medicine Vienna, Vienna, Austria

**Keywords:** Behavioural ecology, Evolution

## Abstract

The ability to learn from experience can improve Darwinian fitness, but few studies have tested whether sexual experience enhances reproductive success. We conducted a study with wild-derived house mice (*Mus musculus musculus*) in which we manipulated male sexual experience and allowed females to choose between (1) a sexually experienced versus a virgin male, (2) two sexually experienced males, or (3) two virgin males (n = 60 females and 120 males). This design allowed us to test whether females are more likely to mate multiply when they encounter more virgin males, which are known to be infanticidal. We recorded females’ preference and mating behaviours, and conducted genetic paternity analyses to determine male reproductive success. We found no evidence that sexual experience influenced male mating or reproductive success, and no evidence that the number of virgin males influenced female multiple mating. Females always copulated with both males and 58% of the litters were multiple-sired. Females’ initial attraction to a male correlated with their social preferences, but neither of these preference behaviours predicted male reproductive success – raising caveats for using mating preferences as surrogates for mate choice. Male reproductive success was predicted by mating order, but unexpectedly, males that copulated first sired fewer offspring.

## Introduction

Mate choice is the differential mating of females as a result of mating preferences^1^ and it can involve processing and integrating information from multiple sensory modalities and other demanding cognitive tasks^2^. There is growing interest in determining how learning might influence sexual selection and vice versa^3,4^. It is unclear why learning has not received more attention in sexual selection research, but perhaps it is because experience has long been viewed as a potential source of error that needs to be controlled. Mate choice studies are often conducted with sexually inexperienced virgins to remove the possible confounding effects of experience. It is important to investigate how learning influences mate choice, not only to better understand the underlying proximate and cognitive mechanisms, but also to obtain ecologically relevant measurements of the functional consequences of mate choice. There have been many studies to determine how age influences a male’s sexual attractiveness and mating success^5^, and though older males often have an advantage^6^, surprisingly little is known about the role of sexual experience – as previous mating experience is usually controlled or not measured (but see^7^). If learning is experimentally controlled, then the direction and strength of sexual selection could be under-estimated or misinterpreted.

Sexual experience can potentially affect male mating and reproductive success by altering male behavior, female preferences, or both. First, sexual experience may improve males’ ability to court and persuade potential mates, or to out-compete rivals. Several studies on invertebrates have found that sexual experience improves male courtship and mating success^8–12^, though comparable studies with vertebrate species are lacking. Some suggestive evidence comes from studies in rats (*Rattus norvegicus*)^13^ and laboratory mice (*Mus musculus f. domesticus)*^14^ showing that prior copulation reduces ejaculation latencies. Sexually experienced lab mice show shorter latencies to mount and intromit, and a reduced number of mounts misdirected towards a female’s head compared to inexperienced males^14,15^. Similarly, sexual experience confers greater copulatory efficiency and increased resistance to the effects of various lesions, castration, and stress in rats (reviewed in^16^). Also, the underlying mechanisms explaining how sexual experience might facilitate copulatory behavior have been intensively investigated in rats^17,18^. However, it remains to be tested whether changes in copulatory behavior from sexual experience increases *reproductive success*. Second, sexual experience might alter a male’s attractiveness to females. Females might prefer virgin over sexually experienced males, as the latter impose a higher risk of disease transmission and enhance a female’s risk to mate with sperm depleted males. Indeed, previously mated males have been determined as lower value mates compared to their virgin competitors in various species^19–21^ and female guppies (*Poecilia reticulate*) have been shown to avoid mating with previously mated males^22^. Alternatively, females might prefer sexually experienced males, as those males might be of superior quality. Given that not all males within a population are able to copulate, sexual experience could reflect male quality and females might gain direct or indirect fitness benefits for their offspring in mating with such males. Some studies in invertebrates have reported that females are attracted to and mate preferentially with sexually experienced males over virgins^23,24^. However, little is known about the role of male sexual experience on female mating preferences in any vertebrate species.

House mice (*Mus musculus)* have complex courtship behaviors, including scent-marking with volatile and non-volatile pheromones and emission of ultrasonic vocalizations (USVs), and there are several ways that sexual experience may influence male courtship and mating behavior (pre-copulatory sexual selection). After exposure to a sexually mature female, male mice increase the pulsatile release of testosterone^25^, scent-marking^26^ and ultrasonic courtship vocalizations (USVs) (Zala *et al*. submitted), and they alter copulatory behaviors^15^. Male scent-marking is correlated with their reproductive success, and USV emission is correlated with copulatory success^27,28^. Male olfactory preferences are more pronounced in sexually experienced than inexperienced males^29^, suggesting that sexual experience elevates sexual arousal or motivation. Sexual experience might also enhance male success in sperm competition by increasing sexual arousal or sperm density^30^; alternatively it might reduce their success due to post-mating refractory period and sperm depletion. Female house mice are polyandrous and multiple paternity is common in wild populations (24–30% of litters^31–33^) and even when females can choose their mates under experimental conditions (29–46% of litters^34–37^). There are several possible functions of multiple-male mating in house mice (reviewed in^34^), which include reducing infanticide. Infanticide is very common in mice^38,39^ and virgin males are particularly infanticidal^38–42^. The act of copulation and cues from pregnant females down-regulate infanticidal behavior and initiate parental behavior in males^43^. Male mice reduce infanticidal behavior towards their mates’ offspring – and even other females’ pups – after copulation and cohabitation with a female^43^. Females are therefore expected to mate with virgin males – and show increased multiple-male mating – when encountering virgins to reduce the risk of infanticide. We previously conducted a mate choice experiment with wild-derived house mice (*Mus musculus musculus)* in which females could choose to mate with either one or two males^34^. We found that the rate of multiple-sired litters decreased over repeated trials, suggesting that females become choosier and less likely to mate multiply as they became sexually experienced. Another possibility is that sexually experienced males become more successful at pre- or post-copulatory sexual selection. However, no study to our knowledge has investigated whether female multiple mating depends upon the sexual experience of potential mating partners. Also, we are unaware of any study that has investigated whether sexual experience influences male reproductive success, either through pre- or post-copulatory mechanisms.

In this study, we experimentally manipulated the sexual experience of wild-derived male house mice (*Mus musculus musculus)*, and we determined the effects on their mating and reproductive success in a mate choice experiment in which direct, male-male interactions were controlled. We manipulated male sexual experience by allowing males to successfully mate with a female, and then we compared the mating and reproductive success of the sexually experienced males with virgin control males in a mate choice experiment where females were allowed to choose between (1) a sexually experienced male versus a virgin male; (2) two sexually experienced males; or (3) two virgin males. This design allowed us to test whether females increase multiple-male mating when they encounter more virgin males. We expected that male sexual experience would influence their mating and reproductive success, and in particular, we expected that females would preferentially mate with virgin males, or be more likely to mate multiply when both males are sexually inexperienced. Alternatively, virgin males may have a lower rate of mating success when competing against experienced males due to their lack of experience. We directly observed mating behavior and conducted genetic paternity analyses to quantify male reproductive success. Female house mice show preferences for a variety of male traits; however, there is surprisingly little known about how female *mating preferences* influence male reproductive success (*mate choice*)^27,36,44^. Therefore, we assessed various measurements of female social preferences and conducted a detailed path analysis to determine whether and how female social preferences translated into mate choice and male reproductive success.

## Methods

### Ethical statement

This study has been discussed and the protocols have been approved and were in accordance with ethical standards and guidelines in the care and use of experimental animals of the Ethical and Animal Welfare Commission of the University of Veterinary Medicine, Vienna (Austria) (Ref. no.:19/07/97/2012) in accordance with Good Scientific Practice guidelines and national legislation.

### Experimental animals and housing

Experimental animals were offspring (F1) of wild-caught house mice (*Mus musculus musculus*) that were trapped at eight locations within a 500 m radius in Vienna, Austria (48°12′38″N, 16°16′54″E). Wild-caught mice were assigned to 28 breeding pairs (crossed between trapping sites) and the F1 offspring were weaned at 21 ± 1 days and subsequently housed individually in type II mouse cages (26.7 × 20.7 × 14 cm) containing wood chips (ABEDD), cotton nesting material (BIOSCAPE) and a cardboard tunnel as shelter. Food (Altromin rodent diet 1324) and water were provided *ad libitum*. Animals were kept at a room temperature of 22 ± 1 °C and a 12:12 h light:dark cycle was maintained. At weaning all animals were ear-punched for individual identification and tissues were stored at −20 °C for genetic paternity analyses. A total of 60 females and 120 males were used for this study and animals were between four to eight months old when the experiment began.

### Experimental treatments: manipulating sexual experience

We assigned individual males to ‘competing pairs’ in three different experimental groups consisting of either (1) one sexually experienced and one virgin male (n = 20 pairs); (2) two sexually experienced males (n = 20 pairs); or (3) two virgin males (n = 20 pairs). Males within pairs were unrelated to each other and matched for age ( ≤ 35 d) and body mass ( ≤ 0.6 g). To manipulate male sexual experience, we housed experimental males with an unrelated and unfamiliar female in a type IIL mouse cage (36.5 × 20.7 × 14 cm) for 18 days (‘sexually experienced’, n = 30). Sexual experience was confirmed when females became pregnant (92% of pairs produced a litter). Control males were kept under the same conditions, except that they were housed individually and only received female odour stimulation (fresh female-soiled bedding, pool of bedding from seven to ten sexually mature females that were unrelated to each other and to the control males) every 5 d during the 18 d treatment phase (‘sexually inexperienced’, n = 30). Odour stimulation was performed to sexually motivate control males, since we aimed to assess differences in mating experience rather than differences in sexual arousal. Housing individual subjects separately allowed us to have independent replicates and avoid pseudo-replication that is commonly found in studies on mice. After the treatment phase, all males received a collar. Collars (2.5 mm wide plastic cable tie with two 2.5 cm long wires attached) were gently fastened around the neck of the animals and restricted males from leaving their arena, and allowed us to prevent female harassment and direct male-male interactions (see ‘Mate choice assay’ below). Males were provided 1 d to become familiar with the device before their release into the mate choice assays.

### Mate choice assay

**Figure 1:**
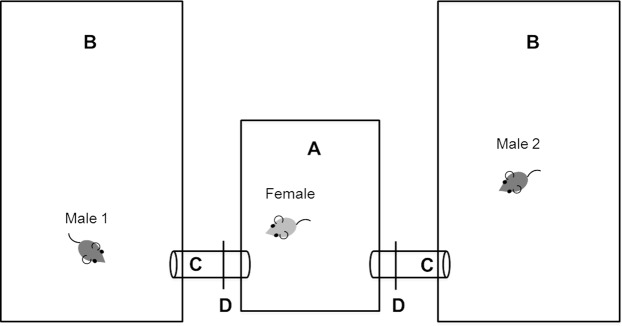
Mate choice assay. Females were placed in a central cage (**A**), males were placed in separate cages at opposite sides of the central cage (**B**), and females could move freely between the three cages, which were connected with tubes (**C**) once the partitions (**D**) were opened. Males had special collars that prevented them from entering the tubes.

Sixty sexually experienced females that produced one litter prior to the experiment were assigned to one of the three experimental groups (n = 20 in each group) in which they could choose to mate with either one or both males. Experimental females were unfamiliar and unrelated to the males. The mate choice arena was composed of three compartments: one central female compartment (type II mouse cage, 26.7 × 20.7 × 14 cm), which was connected to two different ‘male compartments’ on opposite ends (type III mouse cages, 42.5 × 26.6 × 15.5 cm) (Fig. 1). All compartments contained wooden bedding (ABEDD), cotton nesting material (BIOSCAPE), food (Altromin rodent diet 1324) and water *ad libitum*. The compartments were connected with plastic tubes (PVC, 3 cm diameter). Tubes contained metal dividers so that females could move freely between the three compartments only after the dividers were opened. Males were restricted to their own compartments by collars. Males were introduced into the experimental compartments two days before females to allow them sufficient time to scent mark and establish a territory. Also, this additional time provided males time to refill their sperm reserves in case they would have just recently mated during their treatment phase. To synchronize the oestrus cycle of experimental females, they were exposed to male bedding (pool of bedding from five to ten sexually mature males not used in the experiment) two days prior to their introduction^45^. Females were released into the central compartment of the experimental arena at the beginning of the dark phase and after five minutes of habituation, the experiment began by removing the dividers and giving females free access to male compartments. Dividers were opened during the time mice are most active and mating occurs i.e., during the first night females had access to male cages from 6 p.m. to 9 a.m., and from 9 p.m. to 9 a.m. during the following nights. The mice were video-recorded during this time (see ‘Behavioural observations’ below). Experimental mice were monitored daily at 9 a.m. when the dividers were closed and food and nesting material were added whenever necessary. During the daytime, females remained in the central compartment as dividers were closed.

Mate choice trials were terminated after five nights and all mice were returned to the colony. We chose this cut-off time because previous experiments revealed that 95% of females mate within the first five days after pairing under similar conditions, and those that did not become pregnant within this time do not give birth even when provided twice as much time to mate (authors’ unpublished data). Male collars were immediately removed and females were individually housed in type IIL mouse cages (36.5 × 20.7 × 14) with wooden bedding (ABEDD), cotton nesting material (BIOSCAPE) and a plastic mouse house (TECNIPLAST) to give birth under controlled conditions. Reproductive success (litter size and pup body mass at weaning) was measured and genetic paternity analyses were conducted to determine reproductive success. Due to space limitations, the mate choice trials were run in four blocks (15 females were tested per block). All trials within a block were isolated from each other by wooden walls (0.8 m high). The number of experimental groups was balanced between blocks and male assignment to their respective compartment was counterbalanced for their sexual experience status between trials. Female body mass was measured shortly before they were released into the experiment. Male body mass was taken directly before and at termination of the treatment phase and at the end of the experiment when they were returned to the colony.

#### Behavioural observations

Mate choice trials were video recorded (IP cameras, D-Link DCS-3710) and illumination was provided by red lights (Philips RED TL-D 36 W) during the dark cycle. Red light tubes were centrally fixed above the experimental setups and the cameras were operated through a computer (D-ViewCam Version 3.0) located outside of the experimental room. Video analyses were performed in Oberver XT (*Noldus*) and observers were naïve about the males’ sexual experience. Within the first three hours of the experiment we determined female mating preferences, which included (1) female initial attraction towards a male and (2) female social preference for a male. Female *initial attraction* to males was measured as the order and latency to enter a male’s cage and female *social preference* was defined as the total time spent inside a male’s compartment within the three hours observation period. One of the 60 females was excluded from the analysis, as one of her potential mates became sick during the experiment. Mating behaviour was analysed for those females that gave birth after the experiment (48/59), and for this group, the entire 12 h observation period of the mating night was analysed. A *mating event* (*copulation)* was scored each time a male mounted a female and exhibited pelvic thrusts. House mice mount with and without intromission^14^, which are difficult to distinguish without fine-resolution video recordings, and therefore, our definition is a composite measurement of both. The following parameters were analysed for each female: (1) latency to be mounted (mating latency), (2) number of mountings (*mating frequency*); (3) duration of copulations (*mating duration*) and (4) the first and last male a female copulated with. From video recordings it was not always possible to tell whether matings included ejaculations.

#### Genetic paternity analyses

Genetic paternity analyses were conducted to determine the reproductive success of individual males and to assess the rate of single and multiple sired litters. Ear punch samples (or other tissues samples from pups) were used for extracting DNA with a proteinase K/isopropanol protocol^46^. In total, 316 offspring and 144 adults (48 mothers and 96 potential fathers) were genotyped at 4 to 15 microsatellite loci (D11Mit150, D9Mit34, D10Mit20, D17Saha, D9Mit135, D7Mit227, D1Mit456, D15Mit16, D5Mit25, D19Mit39, D2Mit252, D6Mit138, D17Mit21, D2Mit380, D1Mit404, see Mouse Microsatellite Data Base of Japan) using a Multiplex PCR Master Mix (QIAGEN Multiplex PCR Kit). All 15 markers are polymorphic and located on 11 different chromosomes. Amplification mixes (each 10 µl) were subjected to an initial denaturation at 95 °C for 15 min, followed by 30 cycles of denaturation at 94 °C for 30 s, annealing at 55 °C for 90 s and elongation at 72 °C for 60 s, final elongation was followed at 72 °C for 10 min. Each amplification mixture contained 1 µl of extracted DNA (concentration 100–120 ng/µl), 5 µl of QIAGEN Multiplex PCR Master Mix and different volumes of up to 7 primers (each 10 µM), filled up to a reaction volume of 10 µl with HPLC-purified water. Initially, PCR products were checked for successful amplification by electrophoresis on 1% or 2% agarose gels containing TBE buffer and GelRed^TM^ Nucleic Acid Gel Stain (Biotium). Mixtures of 1 µl diluted amplification product (dilution 1:15–1:40), 8.5 µl Hi-Di^TM^ Formamide (Applied Biosystems) and 0.5 µl of an inhouse prepared ROX size standard^47^ were denatured at 96 °C for 5 min and immediately chilled on ice for the following fragment analysis carried out by capillary electrophoresis on an automated DNA sequencer (Applied Biosystems 3130*xl* Genetic Analyzer). For allele scoring GeneMapper^®^ Software Version 4.0 from Applied Biosystems was used. Paternities were assigned by complete exclusion on at least two microsatellite loci; however paternities were also confirmed with a 99% confidence (dam-sire-offspring relationship) by the software CERVUS 3.0.3^48^.

### Statistical analyses

To test whether the rate of multiple paternity was influenced by the experimental treatment we ran a generalized linear mixed model (GLMM) with a binomial distribution and a logit link function. Paternity (single or multiple) was included as the dependent variable, experimental treatment as fixed factor and litter size as a random factor, as the likelihood of detecting multiple paternity increases with litter size. To test whether male sexual experience influenced male reproductive success, we applied a GLMM with a binomial distribution and a logit link function and included the number of offspring sired by each male as the dependent variable, litter size as the binomial denominator, sexual experience status as fixed factor and male pair as a random factor. To test whether male sexual experience affected the latency to mate or the mating duration we performed general linear mixed models (LMM). To test the effect of sexual experience on mating frequency, we applied a GLMM with a Poisson distribution. To test whether male sexual experience affected female social preferences (time spent with males) or the likelihood of first visit (yes or no), we run a LMM and a GLMM with a binomial distribution and a logit link function respectively. We included male pair as random factor in all models as males within pairs were not independent from each other. When assessing the effects of male sexual experience we only included data from the mixed treatment group. We verified that model assumptions were fulfilled in determining model residuals and investigating homoscedasticity of data. If model assumptions were not met we transformed data.

**Figure 2:**
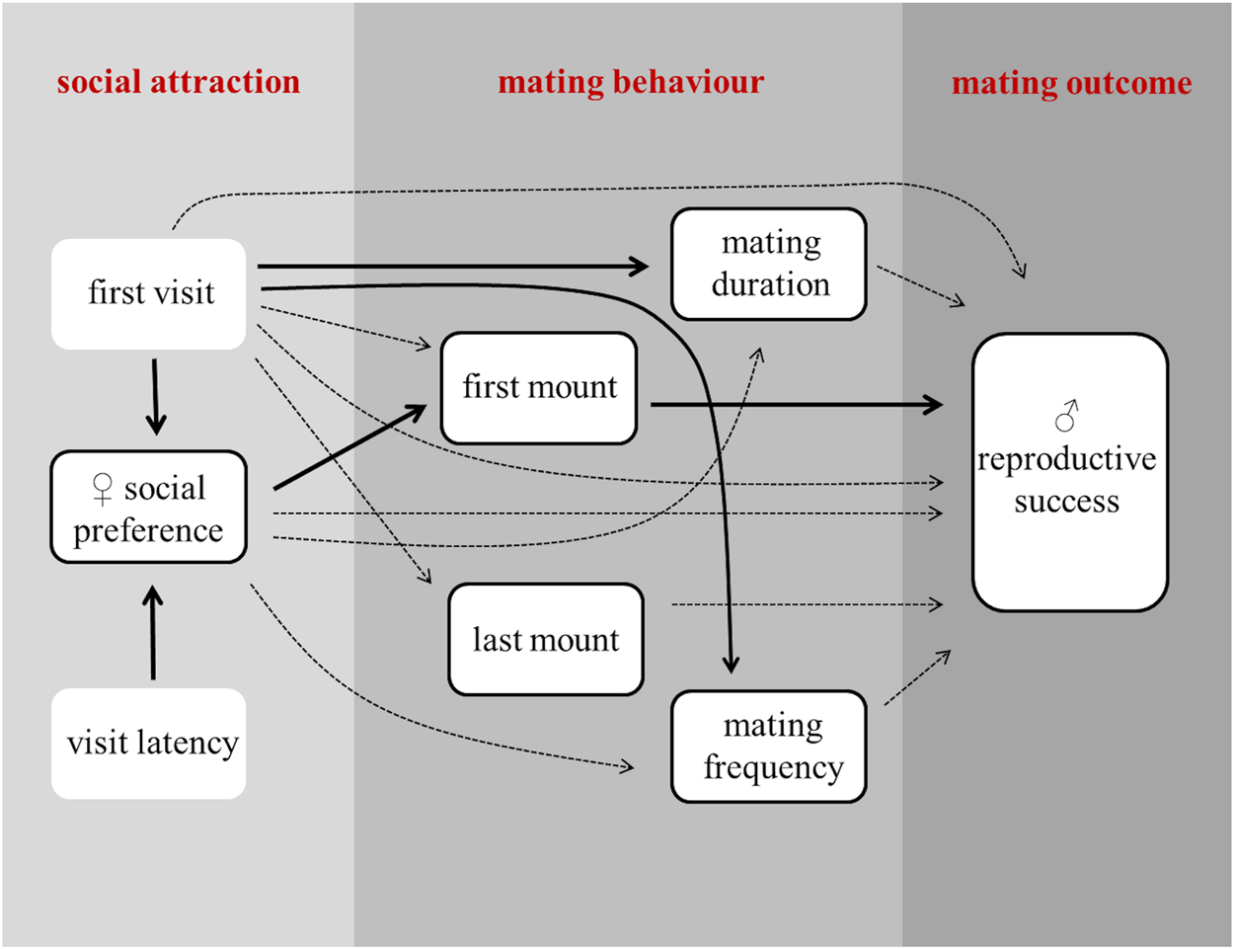
Path diagram showing the recorded behaviors and their effects on specific traits of interest (framed fields). The investigated relationships and the direction of the predicted effects are represented by arrows. Significant relationships are shown in solid bold lines, whereas non-significant ones are depicted with dashed lines.

To examine which factors influence social and mating behavior, and to test how these traits were related to each other and affected reproductive outcomes, we conducted a path analysis (PA). In general, path analyses allow testing the interrelationships among measured variables and how they directly and indirectly affect specific variables of interests. We included female initial attraction, social preference, mating behavior and male reproductive success into the path analysis according to the *a priori* designed path diagram (Fig. 2). To evaluate the relative importance of each path in the diagram, we calculated the standardized partial regression coefficients (path coefficients). Path coefficients estimate the strength of relationships between variables. Effects can either be direct (involving only one step from the trait to the observed outcome) or indirect (involving more than one step). Here we only tested direct effects and applied the path analyses on the whole data set. Male reproductive success was defined as the proportion of offspring sired within the litter, and we applied an arcsine square-root transformation on this variable before using it for statistical calculations.

All statistical analyses were performed using ‘R’ version 3.5.3^49^. We implemented the generalized linear mixed effects models and the general linear mixed effects models using the ‘glmer’ function in the ‘lme4’ package and the ‘lme’ function in the ‘nlme’ package respectively. We implemented the path analysis using the ‘sem’ function in the ‘lavaan’ package.

## Results

**Figure 3:**
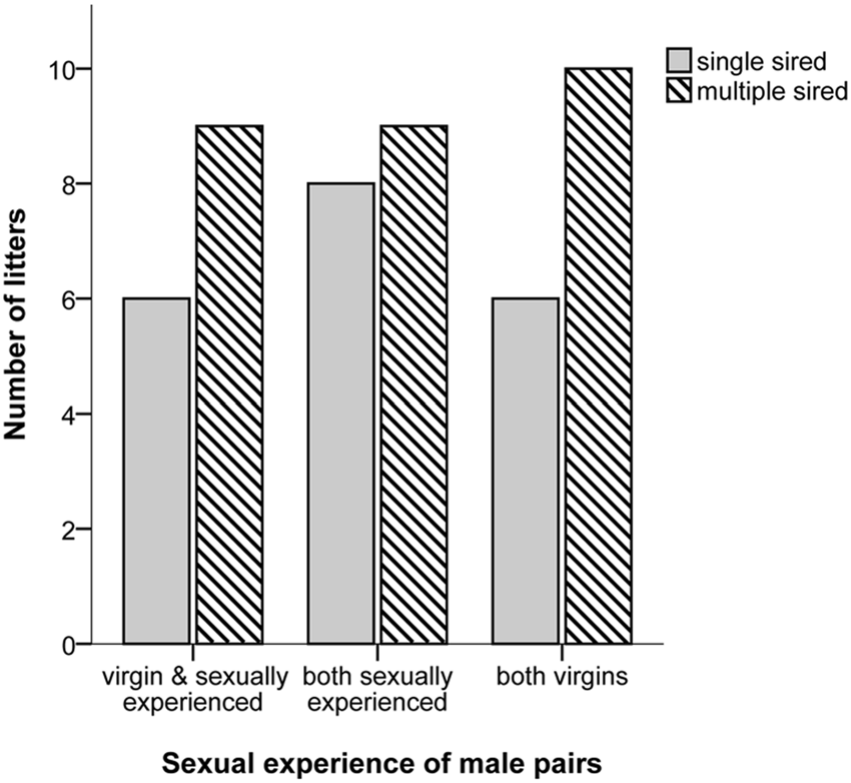
The number of single and multiple sired litters according to males’ sexual experience.

**Figure 4:**
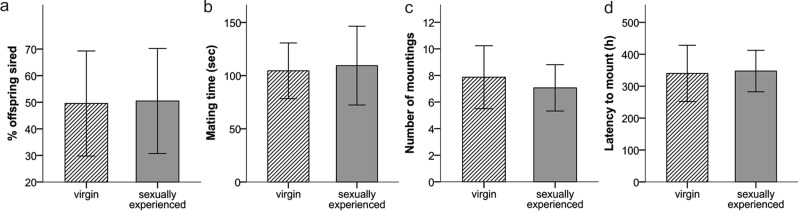
Reproductive success and mating behavior of competing males that differed in their sexual experience status. (**a**) Mean proportion of offspring sired within a litter by virgin versus sexually experienced males. (**b**) Mean mating duration of virgin versus sexually experienced males. (**c**) Mean mating frequency of virgin versus sexually experienced males. (**d**) Mean latency to mate during the mating night of virgin versus sexually experienced males. Error bars show the ±95% CI.

Most of the females (48/60 or 80%) mated and produced a litter and the likelihood of producing a litter did not differ between experimental treatment groups (χ² = 0.272, p = 0.873). Among those females that reproduced, the experimental treatment had no effect on female mate choice, as all females (48/48) copulated with both males (i.e., male were observed to mount and copulate, though this does not mean that ejaculation occurred). Although all females mated multiply, only 58% (28/48) of the litters were multiple sired, and the experimental treatment had no influence on the rate of multiple paternity (GLMM: z = −0.143, β = −0.105, SE = 0.738, P = 0.886, N = 48 pairs) (Fig. 3). When females could choose between two males that differed in their sexual experience status, the proportion of offspring sired from sexually experienced versus virgin males did not differ (GLMM: z = 0.984, β = 0.278, SE = 0.282, P = 0.325, N = 15 pairs) (Fig. 4a). Similarly, we found no evidence that male sexual experience affected mating duration (LMM: t = 0.129, β = 0.119, SE = 0.921, P = 0.899, N = 15 pairs) (Fig. 4b), mating frequency (GLMM: z = −0.804, β = −0.107, SE = 0.133, P = 0.421, N = 15 pairs) (Fig. 4c) or latency to mate (LMM: t = 0.720, β = 19.133, SE = 26.563, P = 0.483, N = 15 pairs) (Fig. 4d). Also, the time females’ spent with males (LMM: t = −0.469, β = −3.978, SE = 8.484, P = 0.645, N = 19 pairs) and the likelihood of first visit (GLMM: z = −0.324, β = −0.211, SE = 0.650, P = 0.746, N = 19 pairs) were not affected by male sexual experience.

**Figure 5:**
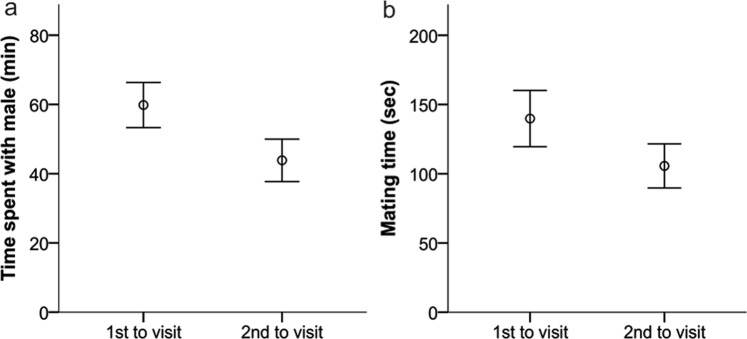
(**a**) Mean time spent with males that a female visited first compared to second. (**b**) Mean time spent copulating with males that a female visited first compared to second. Error bars show the ±95% CI.

**Figure 6:**
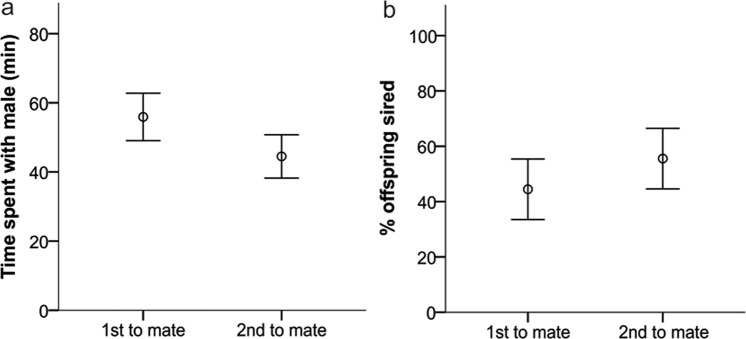
(**a**) Mean time spent with males that a female copulated first versus second with. (**b**) Proportion of offspring sired within a litter from males that copulated first versus second. Error bars show the ±95% CI.

Nevertheless, the path analyses of female behavior provided several interesting results. First, female latency to enter a male’s cage significantly predicted their social preferences: the sooner a female entered a male’s cage, the more time she spent with this particular male overall (PA: z = −2.073, β = −0.011, SE = 0.005, P = 0.038). Females also spent significantly more time with the males they visited first compared to second (PA: z = 2.158, β = 0.177, SE = 0.082, P = 0.031) (Fig. 5a). Second, females mated significantly longer with the first males that they visited (PA: z = 2.367, β = 0.553, SE = 0.234, P = 0.018) (Fig. 5b), but they were not more likely to copulate first with those males (PA: z = 0.911, β = 0.095, SE = 0.104, P = 0.362). Third, female social preferences predicted the likelihood of first copulation: females were more likely to copulate first with those males they spent more time with (PA: z = 2.061, β = 0.005, SE = 0.002, P = 0.039) (Fig. 6a). However, female social preferences did not predict mating duration (PA: z = 1.221, β = 0.370, SE = 0.303, P = 0.222) nor mating frequency (PA: z = 0.538, β = 0.012, SE = 0.022, P = 0.591). Similarly, the last mating event was not related to female social preferences (PA: z = −0.021, β = −0.000, SE = 0.002, P = 0.983). Fourth, neither females’ first visit (PA: z = 0.110, β = 0.014, SE = 0.127, P = 0.912) nor their social preference (PA: z = 0.911, β = 0.002, SE = 0.003, P = 0.362) predicted male reproductive success. Also, there was no effect of mating frequency (PA: z = 0.081, β = 0.001, SE = 0.012, P = 0.936) and mating duration (PA: z = 1.398, β = 0.072, SE = 0.052, P = 0.162) on male reproductive success. Only male mating order had a significant influence on reproductive success, but unexpectedly, males that copulated first with the female sired a lower proportion of offspring within the litter (PA: z = −2.024, β = −0.236, SE = 0.116, P = 0.043) (Fig. 6b). Copulating last had no influence on male reproductive success (PA: β = −0.121, SE = 0.112, z = −1.075, P = 0.283).

## Discussion

Our experiment provides several interesting findings, and our main results include the following. First, we found that male sexual experience had no effect on male mating behavior (latency to mount, mating frequency or mating duration) or reproductive success (number of offspring sired within a litter when accounting for litter size). Surprisingly, virgin males were as competitive as sexually experienced males. There is increasing appreciation of how learning can potentially improve survival and Darwinian fitness, however, our results are a stark reminder of how even complex behaviors, such as courtship and mating, can be fully functional without learning or experience – even in mammalian species^50^. This does not necessarily mean that mating success cannot be improved by learning, and our findings do not rule out this hypothesis. Learning allows animals to change their behavior in response to changes in their environment, and only in predictable environments is it expected to be more adaptive to stick with innate behaviors to reduce the costs of learning^51–54^. Also, as we did not find any effect of socio-sexual experience on male reproductive success, suggesting that individual housing had no detrimental effects on male sexual behavior or competitive ability, contrary to what is often suggested^55–57^ (but see^58^). Second, we found no evidence that females showed more multiple-male mating upon exposure to more virgin males, but this result may have been due to a ceiling effect: females always copulated with both males and such promiscuous mating behavior may function to reduce the risks of infanticide under such conditions. Copulation was not sufficient for males to achieve paternity, since only 58% (28/48) of the litters were sired by both males. This difference in fertilization success may have been due to differences in males’ achieving ejaculation, the competitiveness of their ejaculates (sperm precedence, sperm competition), or both. Paternity is usually skewed in mice^59^. This pattern of paternity may reflect a paternity bias toward the male that copulates closer to the time of ovulation^60^, or alternatively toward the male that has more competitive sperm traits^61^. We are unaware of any studies that have investigated whether or how sexual experience can improve sperm competitive ability, but our data do not support the idea that sexual experience provides an advantage in sperm competition. Third, we found that both measures of female preferences were correlated with each other, but they did not predict male reproductive success. Therefore, the common use of female mating preferences as surrogates for assessing active female mate choice should be treated with caution. We address each of these findings below in more detail.

It is surprising that sexual experience had no effects of male mating behavior or their reproductive success, given the results of previous studies in laboratory house mice and other rodents (see Introduction). Previous studies in invertebrates have shown that sexually experienced males acquired significantly more matings compared to inexperienced rivals in competitive mating assays where males could directly interact with each other^8,9^. Similarly, Preston and Stockley (2006)^62^ reported that male mice adjust their mating behavior in response to their social environment and reduce copulatory stimulation prior to ejaculation, the number of intromissions and mating duration in the presence of a rival. In our experiment, competing males had no contact with each other, and therefore, we cannot exclude the possibility that sexual experience would benefit males when they can directly interact and compete for females. We were unable to clearly discriminate between mountings, intromissions and ejaculations in our video recordings. Nevertheless, we should have been able to detect differences in the latency to initiate mating behavior (time-to-first-mounting event), overall mating duration, and mating frequency between males. Surprisingly, we also found no evidence that male mating behavior influenced reproductive success. Thus, sexual experience provided no fitness benefits for males in pre- or post-copulatory competition. We emphasize that our results may not generalize to more natural conditions. For example, effects from sexual experience may only become apparent when males face a difficult obstacle^63^, such as direct male-male competition or having to court and pursue females in a larger area. In our study we prevented male harassment, and females could evade male mating attempts and disrupt matings by leaving male compartments. Previous studies that have reported differences in male mating behavior placed both sexes in a small arena prior to recording their behavior and did not control for male harassment e.g.^15,64^. Future studies are thus needed to investigate the role of sexual experience on mating behavior and reproductive success under more natural conditions.

We found no evidence that females preferred to mate with virgin males or that they increased their multiple-male mating upon encountering one or more virgin males. The act of mating has been reported to reduce male infanticidal behavior in house mice^38,43^, and therefore females are expected to mate with males that they perceive to be infanticidal. Interestingly, all of the females in our experiment copulated with both males (i.e., both males were observed to mount and copulate, though this does not mean that ejaculation necessarily occurred), which helps explain the high rate of multiple paternity, i.e., 58% of litters. This rate is higher than other laboratory mate choice experiments (e.g. 29–46% of litters^34–37^) and higher than found in wild populations of house mice (24–30% of litters^31–33,59^). We suspect that the high rate of multiple mating in our study was due the females perceiving a risk of infanticide from both males, as they were in close proximity. A previous laboratory mate choice experiment with house mice also found that females invariably copulated with both males when they could choose between a ‘dominant’ and a ‘subordinate’ male^65^, though paternity was not investigated. Again, we emphasize that our results may not apply to studies in larger or otherwise more natural conditions in which females might avoid infanticide by males (but see^36^).

Our analyses of female mate preferences and male reproductive success provided the most important results. As expected, females that were initially attracted to a particular male (first visit), also spent more time with this male, indicating that initial attraction is a reliable measurement of social preference. Females’ initial attraction (first visit) also predicted mating duration and frequency, but not the likelihood of first copulation. Females social preferences (time spent with male), on the other hand, was a predictor of first copulation, but not mating duration or frequency. These results show that only specific measurements of attraction predict mate choice and overall, the results are in line with other studies that report that female social preferences are not always reliable indicators of mate choice^66–68^. In our experiment, female social preference was a good predictor of the likelihood of first copulation and initial attraction was positively correlated with mating duration and frequency, however, neither first visit nor social preference by themselves predicted male reproductive success, and neither did mating duration or mating frequency. Only mating order affected male reproductive success, but unexpectedly, males that copulated first sired fewer offspring. This result is surprising since two studies reported that male mice sire more offspring within a litter when they mate first compared to second^69,70^. The discrepancy in results might be explained by differences in experimental designs: In the studies by Firman & Simmons (2008)^69,70^ estrus females were kept in polyandrous mating regimes where they were sequentially mated with two or three different males that had been assigned to them. Females could not choose between mating partners and a new male was provided after the detection of a mating plug, which ensured that each male ejaculated. In our study, females could choose between mating partners and interestingly, all females went back and forth between male compartments mating repeatedly with both males. This behavior could allow females to evaluate both males, or females used the post-ejaculatory refractory period of one male to mate with the other. Unfortunately, we could not distinguish between mounts with versus without intromissions and ejaculations and thus, differences in results might be explained by differences in the occurrence or timing of ejaculations. Successful ejaculation results in the deposition of a copulatory plug, and previous studies in mice showed that copulatory plugs affect the mating behavior of the second male to mate^71^ and can influence the reproductive success of males mating in this position^71,72^.

Rolland *et al*.^65^ found that female mice – when given a choice between a ‘subordinant’ and a ‘dominant’ male – always copulated with both males, but dominant males were significantly more likely to ejaculate last, suggesting that females bias paternity towards dominant males. However, whether males have last sperm precedence when females can freely choose between mates is not known, as Rolland *et al*.^65^ did not measure male reproductive success, and copulating last was not a good predictor of male reproductive success in our study. In conclusion, our results do not support the hypothesis that sexual experience enhances male mating or reproductive success when females can freely choose between mates. This is the first study to test this hypothesis in a vertebrate species to our knowledge, and future studies are needed that experimentally investigate whether male-male interactions and male-female interactions might be influenced by male sexual experience. Studies are also need to investigate whether sexual and reproductive experience influences female survival or reproduction. Given that females could avoid male mating attempts at any time, our data provide additional support for the hypothesis that multiple mating is a result of active female choice rather than sexual coercion. We also found no evidence to support the assumption that standard measures of female *mating preferences* provide a reliable proxy for mate choice, i.e. reproductive outcomes. Even though female social preference predicted first mating, first mating was surprisingly associated with reduced male reproductive success, suggesting that females can potentially use mating order as a mechanism to bias paternity towards specific males. Even though mate choice has been intensively studied over the past decades only little is known about how mating preferences translate into mate choice. The vast majority of mate choice studies investigated female’s sensory bias or attraction in a brief, one-time presentation of unfamiliar males or male stimuli and considered female responses to work as a proxy for mate choice. However, mate choice depends on individual mating preferences and the extent to which they can be expressed^73,74^ and given the constraints of most experimental assays this assumption has to be validated^75^. Future studies should be aware that female social preferences are not a reliable measurement of mate choice and studies should validate their proxies not to underestimate or misinterpret the role of female choice in sexual selection.

## Data Availability

The datasets generated during and/or analysed during the current study are available from the corresponding author upon request.
